# AD Knowledge Portal, ELITE Portal and Agora: Resources that empower the research community to find, access, and re‐use data and tools related to dementia and ageing

**DOI:** 10.1002/alz70855_101428

**Published:** 2025-12-23

**Authors:** Susheel Varma, Laura M Heath, Jessica Malenfant, Karina Leal, Jessica S Britton, Milan Vu, Solveig K Sieberts, Christine Suver, Alberto Pepe

**Affiliations:** ^1^ Sage Bionetworks, Seattle, WA, USA

## Abstract

**Background:**

To identify disease‐modifying therapies and drug targets for Alzheimer's disease (AD), it is necessary to assess the impact of cellular and molecular dysfunction on disease aetiology. The AD Knowledge Portal (Portal) (https://adknowledgeportal.org), Exceptional Longevity Portal (ELITE) and Agora (https://agora.adknowledgeportal.org) are community‐driven resources that support researchers as they: 1) identify new molecular hypotheses and mechanisms, 2) evaluate hypotheses via independent experimental assessments, and 3) prioritise new molecular mechanisms for therapeutic development.

**Method:**

The Portals and Agora are free, open‐access tools built to maximise therapeutic discovery by enabling researchers to re‐use data from 41,000+ biospecimens collected from 12,000+ individuals across four species and browse or access analytical results related to putative drug targets. The Portals and Agora empower the research community with a trusted repository of data, computational and experimental tools and potential gene targets to test target hypotheses and therapeutics. The portals are also integrated with external Trusted Research Environments (TREs) like CAVATICA and integrate the portals with Terra and AD Workbench.

**Result:**

The Portal supports research outputs from 55 grants (including the Accelerating Medicines Partnership‐Alzheimer's Disease), each generating data and computational/experimental tools related to dementia and ageing. Utilizing samples from brain banks, longitudinal cohorts, and model systems, available data spans 55+ assays, including genomics, metabolomics, imaging, cognitive assessments, and more. Model systems in the Portal include iPSC‐derived cell types and organoids and novel Late‐Onset AD mouse models based on gene targets identified from human data. These mouse models are available with no limitation on use. The Portal also features a cloud‐based analytical workspace providing access to preconfigured computational resources to process, integrate, and analyse data. A subset of the processed data is also presented in Agora, a visual results explorer that compiles evidence of genes' association with AD. Agora hosts a list of nominated targets and presents the results of omics analyses generated from Portal data. It also includes a catalogue of details and results from targeted validation studies.

**Conclusion:**

The AD Knowledge Portal, the ELITE Portal and Agora offer the research community an accessible, rich data source, tools, and results.

